# Epidermal Growth Factor Receptor Mutation Status and Response to Tyrosine Kinase Inhibitors in Advanced Chinese Female Lung Squamous Cell Carcinoma: A Retrospective Study

**DOI:** 10.3389/fonc.2021.652560

**Published:** 2021-04-02

**Authors:** Qing Chang, Huiping Qiang, Jialin Qian, Yuqiong Lei, Jiahuan Lu, Hui Feng, Yiming Zhao, Baohui Han, Yanwei Zhang, Tianqing Chu

**Affiliations:** ^1^ Department of Pulmonary, Shanghai Chest Hospital, Shanghai Jiaotong University, Shanghai, China; ^2^ Department of Emergency, Shanghai Chest Hospital, Shanghai Jiaotong University, Shanghai, China

**Keywords:** female, lung cancer, squamous cell carcinoma, epidermal growth factor receptor, tyrosine kinase inhibitor

## Abstract

**Background:**

The frequency of epidermal growth factor receptor (EGFR) mutations and the efficacy of tyrosine kinase inhibitor (TKI) in Chinese female patients with lung squamous cell carcinoma (SCC) are unknown. This study was designed to investigate the incidence of EGFR mutations and the role of targeted therapy in advanced Chinese female lung SCC patients.

**Methods:**

Advanced female patients diagnosed with lung SCC at the Shanghai Chest Hospital between January 2013 and December 2018 were retrospectively analyzed.

**Results:**

A total of 4223 advanced lung SCC patients were screened, and there were 154 female lung SCC patients who had underwent EGFR mutation detection. Positive EGFR mutations were found in 29.9% (46/154) of female lung SCC patients, including twenty-three 19del mutation (14.9%), twenty-one 21L858R mutation (13.6%) and other mutations (1.4%, 21861Q and 20ins). For 45 EGFR positive mutation female SCC patients, the median progression-free survival (PFS) of patients who received EGFR-TKI therapy (n=38) was 8.0 months (95% CI, 5.4-10.7 months), which was significantly longer than patients who were treated with chemotherapy (8.0 vs. 3.2 months, p=0.024), and the median overall survival (OS) was also longer (24.9 months vs. 13.9 months, p=0.020). The objective response rate (ORR) was 44.7% (17/38), and the disease control rate (DCR) was 81.6% (31/38). For 105 female SCC patients with EGFR negative mutation, the median OS was 18.6 months (95% CI, 14.2-22.9 months) and it was no different from that of EGFR positive mutation patients (18.6 vs. 22.8 months, p=0.377).

**Conclusion:**

For advanced Chinese female lung SCC patients with EGFR positive mutations, targeted therapy could confer longer PFS and OS than chemotherapy, but the survival was similar with patients who were negative EGFR mutations.

## Background

Lung cancer is the leading cause of cancer incidence and mortality both in the world and China (1–3). Approximately 75% of these patients have lost the opportunity for surgery at the time of diagnosis (4). With the development of molecular detection and targeted drug research and development, molecular targeted therapy has become the indispensable treatment for advanced non-small cell lung cancer (NSCLC) patients. Among all the driving gene mutations, the most common one is epidermal growth factor receptor (EGFR) gene mutation, especially for the patients in east Asia, non-smoking, women and adenocarcinoma (5). Compared with advanced lung adenocarcinoma patients, the EGFR mutation rate of patients with lung squamous cell carcinoma (SCC) is much lower, about 4.2%-23.8% only (6–13). So most patients with lung SCC have no chance to get benefit from EGFR tyrosine-kinase inhibitors (TKIs). However, it was found in the subgroup analysis of several studies that among all lung SCC patients, the EGFR mutation rate in female patients seemed to be higher, about 14.4% to 33.3% (6, 10, 11, 14). But the sample size of female SCC in these studies were very small, and at present there was no specific study on EGFR mutation status in female lung SCC.

Several prospective clinical trials have made EGFR TKIs become standard first-line treatment for EGFR mutation-positive NSCLC patients (15–23). However, the number of female lung SCC patients in these studies was still very small, or even none. Previous respective studies showed that lung SCC patients with sensitive EGFR mutations could obtain clinical benefits from EGFR-TKIs (9, 13, 24). While in a study of Turkish female lung SCC patients with EGFR positive mutation, the response to gefitinib was not good (14). Therefore, the efficacy of EGFR-TKIs for female lung SCC patients with sensitive EGFR mutations remains unclear.

In this study, we summarized clinical data of female lung SCC patients treated in Shanghai Chest Hospital, in order to investigate the incidence of EGFR mutations and the efficacy of EGFR-TKIs in Chinese female lung SCC patients.

## Materials and Methods

### Patients

We retrospectively collected and analyzed the data of patients who had been confirmed advanced lung SCC at Shanghai Chest Hospital between January 2013 and December 2018. All information of these patients was identified from our electronic medical records system. The patients who met the following criteria were included in the study: (1) pathologically or cytologically confirmed pure lung SCC; (2) confirmed with a diagnosis of stage IIIB or IV (the stage was performed according to the eighth edition of the TNM classification for NSCLC) (25); (3) detected the EGFR mutation status. The baseline clinical characteristics included age, smoking history, long history of exposure to secondhand smoke (>2h every day at home or indoors for at least ten years), long history of exposure to cooking oil fumes (the total cooking history > 50 dish-years, including stir frying, frying and deep frying), EGFR mutation status, tumor location, clinical stage, metastatic sites and sample type (26).

The study was approved by the independent ethics committee of Shanghai Chest Hospital, and all the patients signed informed consent before therapy.

### EGFR Mutation Detection

All the patients have been detected the EGFR mutation using tumor tissue samples (from small biopsy or operation). The DNA was extracted from formalin-fixed paraffin-embedded (FFPE) tissue sections according to the manufacturer’s protocol (QIAamp DNA FFPE Tissue Kit, Qiagen, Hilden, Germany).

Two methods were used to detect EGFR mutation, the amplification refractory mutation system-polymerase chain reaction (ARMS-PCR) or Next-generation Sequencing (NGS). ARMS-PCR was used on the DxS EGFR mutation test kit (Manchester, UK). Tumor content in the specimen that had undergone conventional gene test was evaluated by a pathologist. Specimens with more than 5% tumor content were eligible for NGS. The quantity and quality of DNA was assessed by Qubit^®^dsDNA HS Assay Kit on Qubit^®^3.0 Fluorometer (Invitrogen, CA, US). Capture-base targeted sequencing was performed with the Lung core 68 Gene Panel (Burning Rock Biotech, Guangzhou, China). More details of the NGS method can be found in previous reports (27, 28).

### Treatment and Follow-Up

Patients received imaging assessment every 2 months. The response evaluation was assessed according to the Response Evaluation Criteria in Solid Tumors (RECIST guideline 1.1), which defined complete response (CR), partial response (PR), stable disease (SD), progressive disease (PD), objective response rate (ORR) and disease control rate (DCR) (29). The progression-free survival (PFS) for patients received EGFR-TKI therapy was determined from the date of initiation of TKI treatment to the date of disease progression or death. The overall-survival (OS) was defined as the time from the diagnosis date to the date of death or last follow-up visit (October 10, 2019).

### Statistical Analysis

The normal distribution test of continuous variables was carried out, and abnormally distributed continuous variables were expressed as median and further analyzed by the Wilcoxon rank sum test. Categorical variables were described by the means of absolute and percentage numbers and compared with Chi-square test or Fisher’s exact test as appropriate. The survival curves of PFS and OS were estimated according to Kaplan-Meier method and were compared using the log-rank test. ORR and DCR for each group were compared using Chi-square test or Fisher’s exact test when appropriate. Statistical significance was defined as P value < 0.05. All analyses were performed with the SPSS software, version 25 (IBM, Grouponk, NY).

## Results

**Figure 1:**
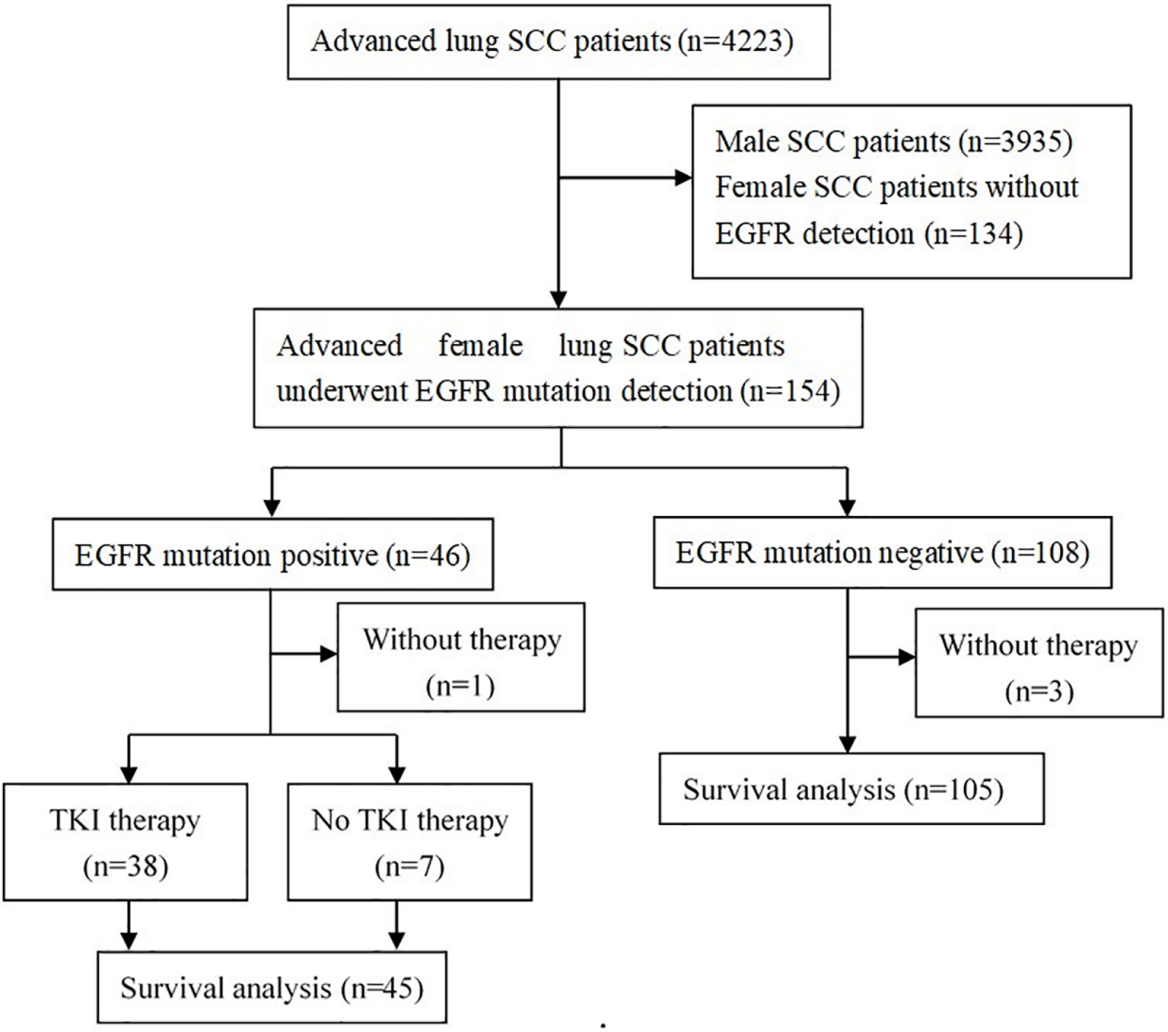
Flow diagram of patients studied. SCC, squamous cell carcinoma; EGFR, epidermal growth factor receptor; TKI, tyrosine kinase inhibitor.

From January 2013 to December 2018, a total of 4223 patients were diagnosed as advanced lung SCC in our hospital, including 288 (6.8%) females and 3935 (93.2%) males, with a male-to-female ratio of 13.7: 1. Of the 288 female advanced lung SCC patients, 154 (53.5%) patients have undergone EGFR mutation detection. The flow chart is shown in **Figure 1**.

### Results of EGFR Mutation Detection

**Figure 2:**
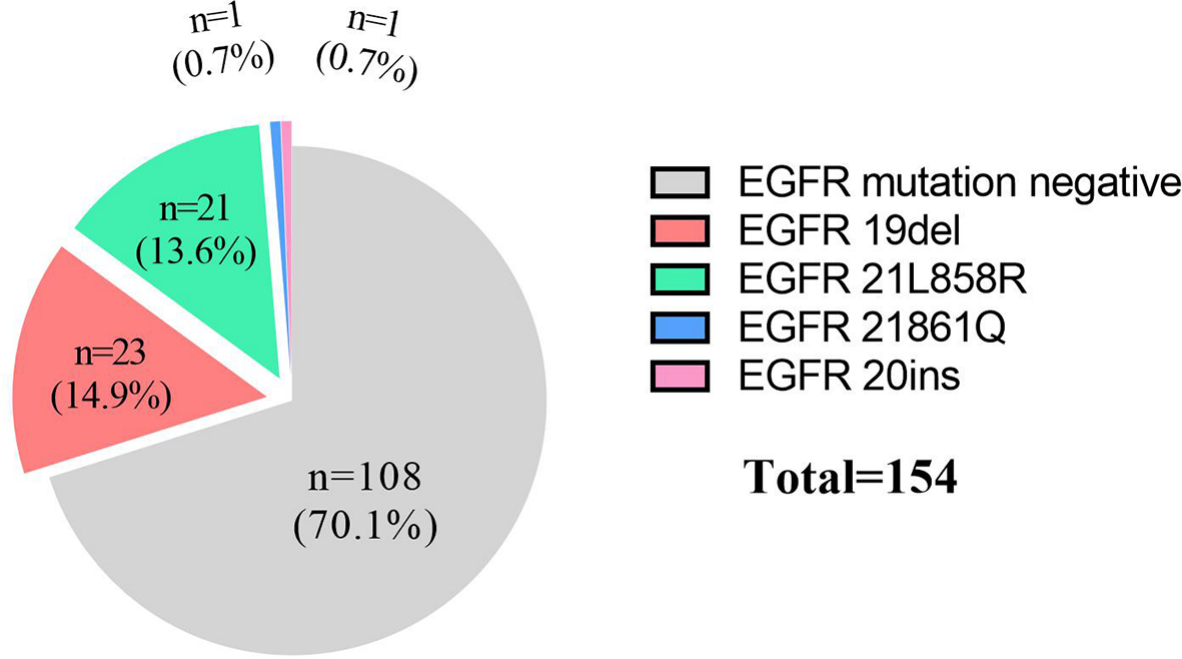
Detailed information about mutations.

For these 154 patients, EGFR mutation rate of advanced female lung SCC patients was 29.9% (46/154), the details as following: 23 patients were 19del (14.9%), 21 patients were 21L858R (13.6%), 1 patient was 21861Q (0.7%) and 1 patient was 20ins (0.7%, **Figure 2**). The positive rates of EGFR detection by ARMS-PCR and NGS methods were 29.9% (40/134) and 30.0% (6/20), respectively. There was no significant difference between them (p=0.99).

**Figure 3:**
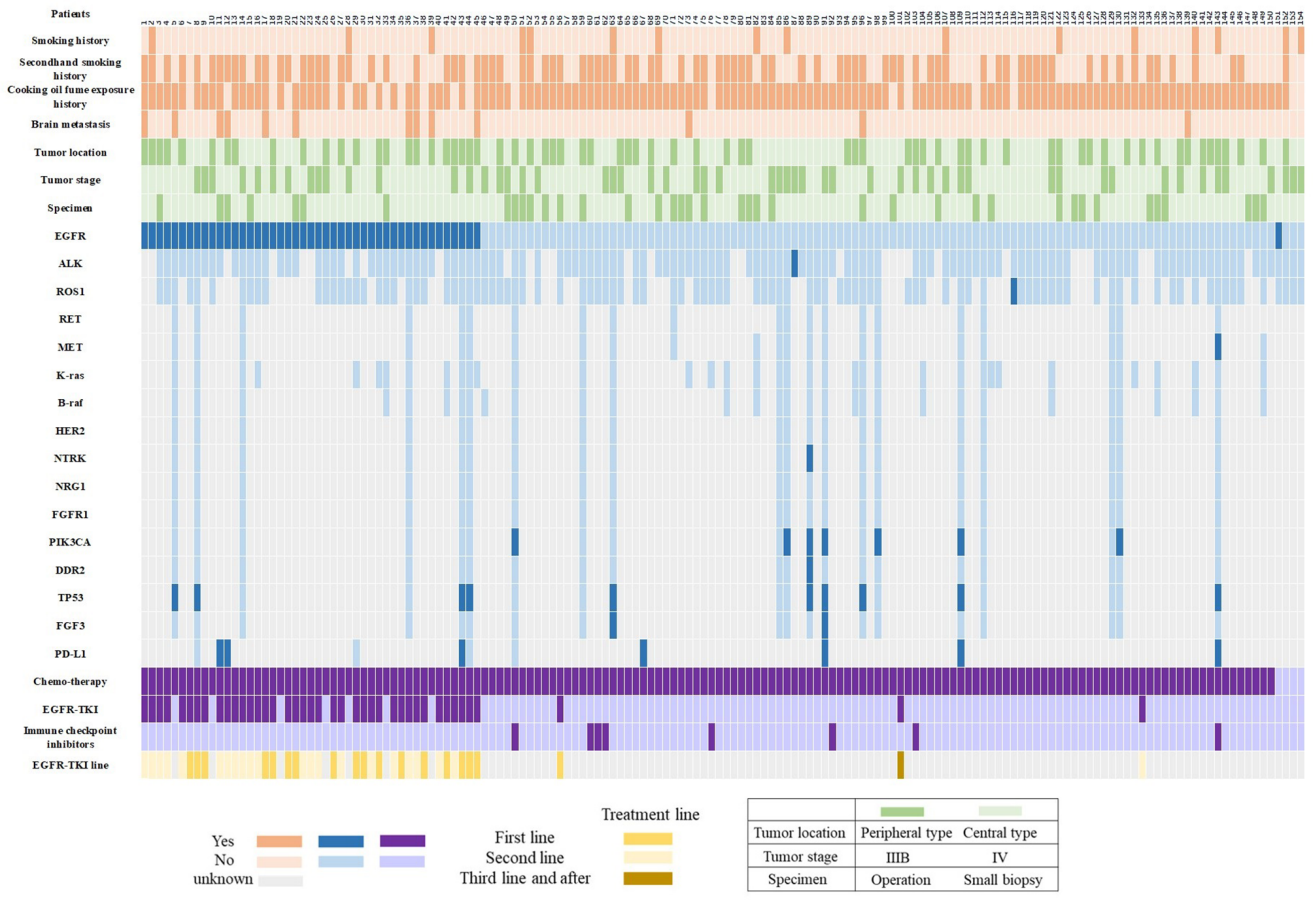
Graphical distribution of the genomic profile. PD-L1, programmed death ligand-1; EGFR-TKI, epidermal growth factor receptor -tyrosine kinase inhibitor.

For patients (n=20) who were genetically tested by NGS method, 19 (90.0%) patients had at least 1 mutation of the 68 cancer-related gene alterations. **Figure 3** shows the frequency of 13 lung cancer driving genes, including EGFR, ALK, ROS1, RET, MET, KRAS, BRAF, HER2, NTRK, NRG1, FGFR1, PIK3CA and DDR2. EGFR mutations were identified in 30% (6/20) of all twenty patients who were genetically tested by NGS method, PIK3CA (35%, 7/20), MET (5%, 1/20), DDR2 (5%, 1/20) and NTRK (5%, 1/20) gene mutations also were found in these patients. In addition to the above driving genes, a total of 5 genes with in review a mutation rate of more than 10% were identified: TP53 (50%, 10/20), FGF3 (15%, 3/20), CDKN2A (10%, 2/20), ATM (10%, 2/20) and CDK4 (10%, 2/20).

### Patient Characteristics

Demographic and baseline clinical characteristics of female SCC patients with EGFR positive or negative mutations were shown in **Table 1** and **Figure 3**. The outcome indicated that for patients with EGFR positive mutations, the percentage of patients with long history of exposure to cooking oil fumes was lower than patients with negative mutations (73.9% vs. 92.6%, p=0.002). The proportion of brain metastasis in patients with EGFR positive mutations was significantly higher than that in negative mutations patients at baseline (21.7% vs. 2.8%, p < 0.001). Other baseline clinical characteristics were balanced between patients with positive and negative EGFR mutations.

**Table 1:** The clinical characteristics of patients.

Characteristics	All patients (n=154)		p-value	Patients with EGFR-mutation and received therapy (n=45)		p-value
	Mutation-positive (n=46)	Mutation-negative (n=108)		EGFR-TKI therapy(n=38)	Chemotherapy(n=7)	
Median age, years	59.5	62.0	0.748	60.0	56.0	0.766
Smoking history			0.395			0.059
Former smoker	3(6.5%)	13(12.0%)		1(2.6%)	2(28.6%)	
Never smoker	43(93.5%)	95(88.0%)		37(97.4%)	5(71.4%)	
Long history of exposure to secondhand smoke			0.642			0.684
Yes	27(58.7%)	59(54.6%)		22(57.9%)	5(71.4%)	
No	19(41.3%)	49(45.4%)		16(42.1%)	2(28.6%)	
Long history of exposure to cooking oil fume			**0.002**			1.000
Yes	34(73.9%)	100(92.6%)		28(73.7%)	5(71.4%)	
No	12(26.1%)	8(7.4%)		10(26.3%)	2(28.6%)	
Mutation status			–			0.272
Exon 19 deletion	23(50.0%)	–		19(50.0%)	4(57.1%)	
L858R mutation	21(45.7%)	–		18(47.4%)	2(28.6%)	
Others	2(4.3%)	–		1(1.7%)	1(14.3%)	
Tumor location			0.341			1.000
Central type	23(50.0%)	63(58.3%)		19(50.0%)	3(42.9%)	
Peripheral type	23(50.0%)	45(41.7%)		19(50.0%)	4(57.1%)	
Tumor stage			0.432			0.659
IIIB	14(30.4%)	40(37.0%)		11(28.9%)	3(42.9%)	
IV	32(69.6%)	68(63.0%)		27(71.1%)	4(57.1%)	
Brain metastasis			**<0.001**			0.642
Yes	10(21.7%)	3(2.8%)		8(21.1%)	2(28.6%)	
No	36(78.3%)	105(97.2%)		30(78.9%)	5(71.4%)	
Specimen			0.060			1.000
Operation	7(15.2%)	32(29.6%)		6(15.8%)	1(14.3%)	
Small biopsy	39(84.8%)	76(70.4%)		32(84.2%)	6(85.7%)	

EGFR, epidermal growth factor receptor; TKI, tyrosine-kinase inhibitor.

Two-sided P value was derived from Wilcoxon rank sum test for continuous variables and from Chi-square test or Fisher’s exact test for categorical variables.

Bold values indicated statistical significance.

### Clinical Outcome/Efficacy

For these 154 patients who had been detected the EGFR status, there were 3 patients with EGFR mutation-negative and 1 patient with positive mutation did not receive any anti-tumor drug. A total of 150 patients (45 positive mutation and 105 negative mutation) were available for the survival analysis in our study (**Figure 1**). For these patients who could be included in survival statistics (n=150), their baseline clinical characteristics are similar to those of all patients (n=154), the detail results were shown in **Supplementary Table 1**.

Of the 45 EGFR mutation positive patients, 38 patients received EGFR-TKI therapy and 7 patients were treated with chemotherapy (**Figure 1**). The baseline clinical characteristics were balanced between patients with TKI therapy and with chemotherapy (**Table 1**). Among the 38 patients with eligible survival data for TKIs, 17 (44.7%) and 21 (55.3%) patients used TKIs as first-line and second-line therapy, respectively (**Figure 3**). Of these patients, 36 received first-generation TKIs (16 gefitinib, 14 icotinib and 6 erlotinib) and 2 received second-generation TKI (afatinib).

**Figure 4:**
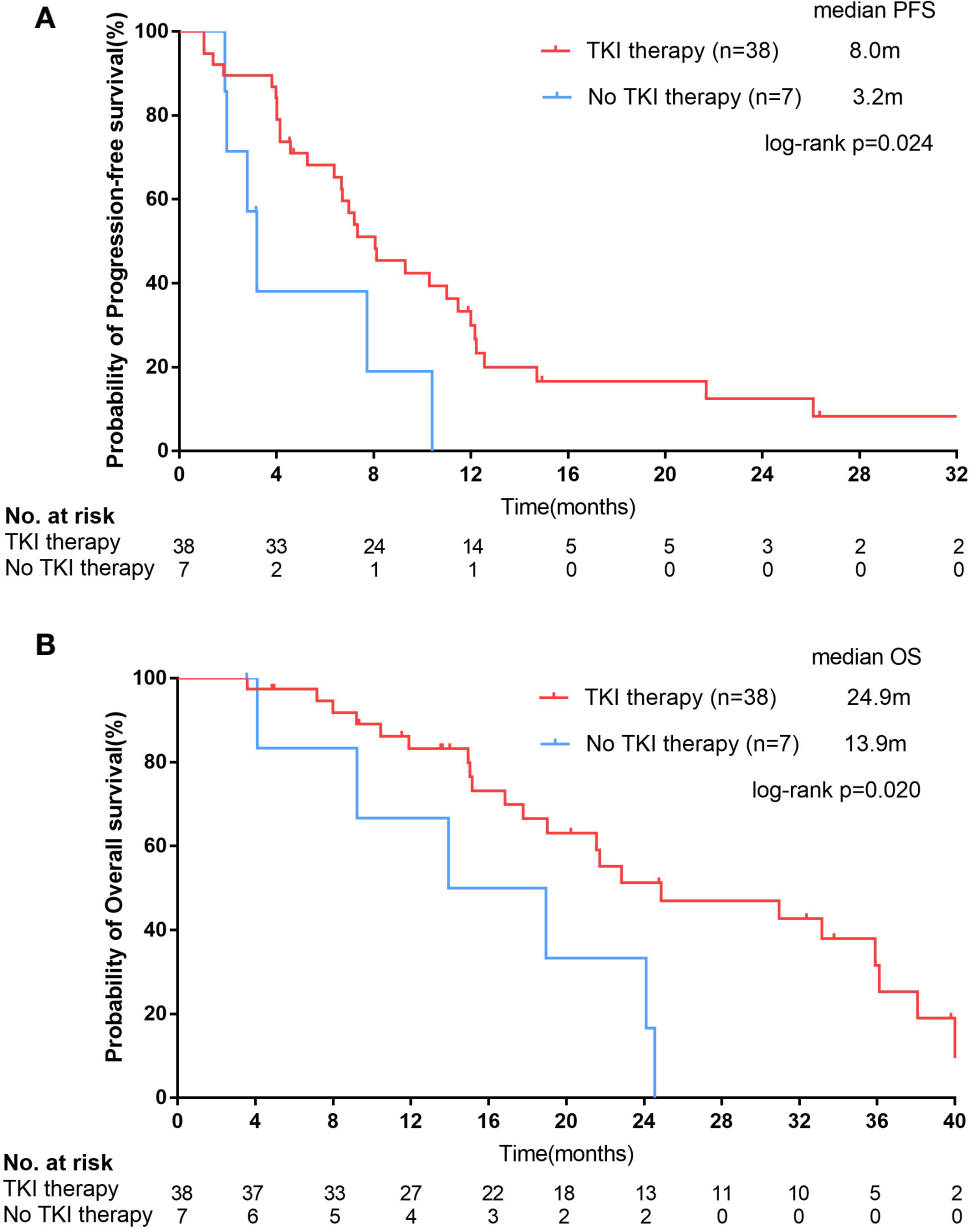
For female squamous cell carcinoma (SCC) patients with epidermal growth factor receptor (EGFR) positive mutations, Kaplan–Meier curves of progression-free survival (PFS, **A**) and overall survival (OS, **B**) stratified by receipt of EGFR tyrosine kinase inhibitor (TKI) therapy.

For 45 EGFR mutation-positive female SCC patients, by the last follow-up, a total of 31 and 6 patients had reached disease progression on EGFR-TKI therapy and chemotherapy, respectively. The median PFS of patients who received EGFR-TKI therapy (n=38) was significantly longer than patients who treated with chemotherapy (n=7; 8.0 months [95% CI, 5.4-10.7 months] vs. 3.2 months [95% CI, 2.4-4.0 months]; p=0.024; **Figure 4A**). Longer OS time was observed in EGFR-TKI group than that in chemotherapy group (24.9 months [95% CI, 11.1-38.6 months] vs. 13.9 months [95% CI, 2.3-25.6 months]; p=0.020; **Figure 4B**). Patients received EGFR-TKI therapy also had higher ORR (44.7%, 17/38) and DCR (81.6%, 31/38). Unfortunately, the difference of ORR and DCR between TKI group and chemotherapy group didn’t reach significance (ORR=14.3%, p=0.137; DCR=71.4%, p=0.430).

**Figure 5:**
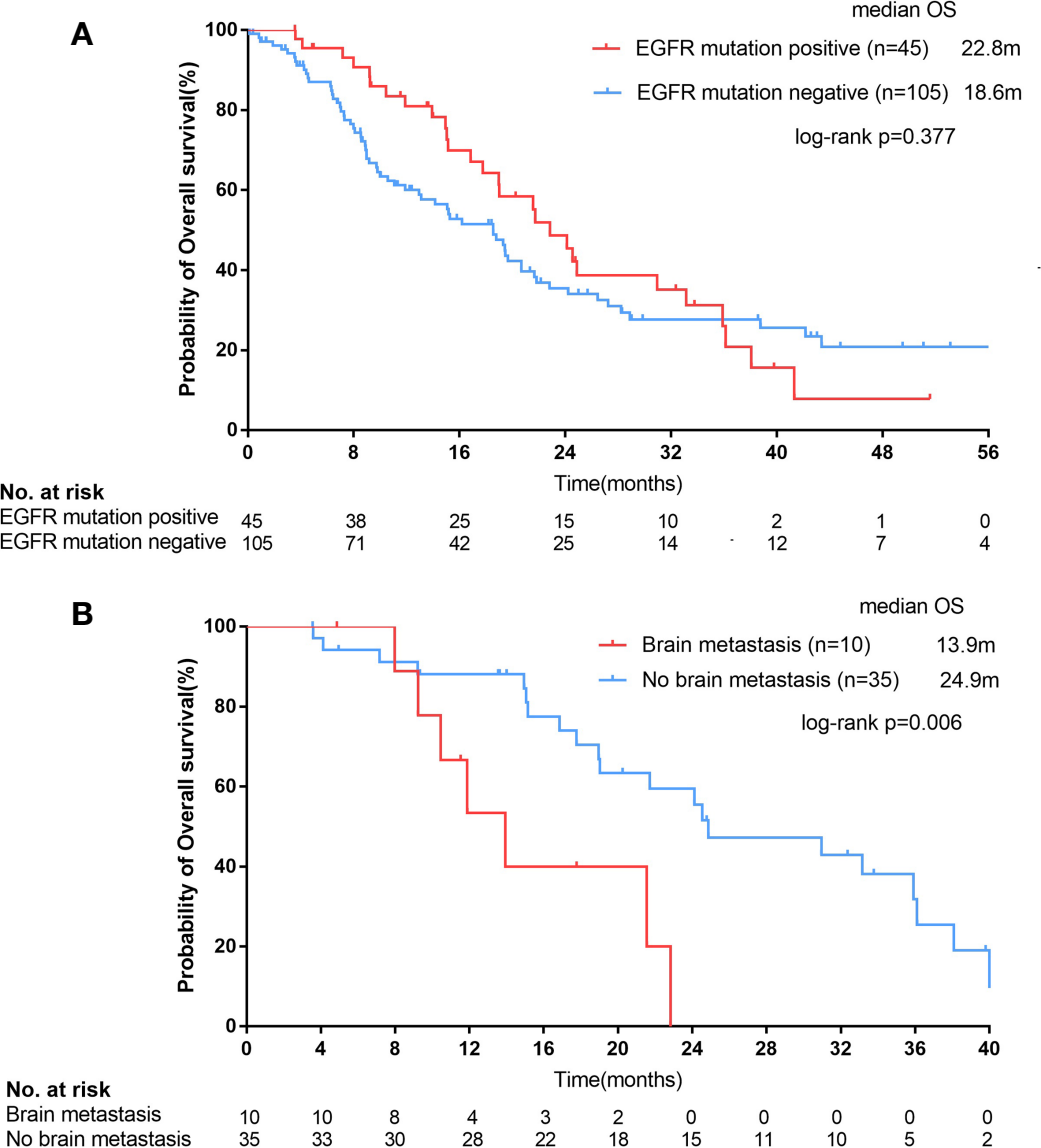
For all female squamous cell carcinoma (SCC) patients, Kaplan–Meier curves of overall survival (OS, **A**) stratified by epidermal growth factor receptor (EGFR) mutation status. For SCC patients with EGFR positive mutations, Kaplan–Meier curves of OS **(B)** stratified by brain metastasis.

In all 150 patients, 45 patients with EGFR mutation had a median survival of 22.8 months (95% CI, 19.0-22.7 months), while 105 patients with negative mutation had a median survival of 18.6 months (95% CI, 14.2-22.9 months), but there was no statistical difference between them (p=0.377; **Figure 5A**).

The median OS of patients with brain metastasis was 11.9 months (n=13, 95% CI, 7.3-16.5 months) in the whole population (13/150), which was shorter than those without brain metastasis (n=137, median OS=19.5 months; 95% CI, 16.6-22.3 months), but the difference was not statistically significant (p=0.126). However, for those EGFR mutation-positive female SCC patients (n=45), the median OS of patients with brain metastasis (n=10) was significantly shorter than those without brain metastasis (n=35; 13.9 months [95% CI, 9.5-18.4 months] vs. 24.9 months [95% CI, 14.6-35.2 months]; p=0.006; **Figure 5B**).

By the follow-up, 37 and 84 patients in the EGFR-positive and EGFR-negative groups had progressed, respectively, and the proportion of patients who sequentially received follow-up treatment was 54.1% (20/37) and 46.4% (39/84, p=0.439), respectively. Among EGFR-positive patients with disease progression and received follow-up treatment (n=20), 8 patients switched to Osimertinib and another 12 patients switched to chemotherapy. For 8 patients who sequentially received Osimertinib therapy, 2 (25.0%, 2/8) of them were positive with acquired EGFR-T790M mutation, 3 (37.5%, 3/8) were negative and 3 (37.5%, 3/8) without re-detection. The median OS of patients who received and didn’t receive the third-generation TKI treatment was 30.0 and 21.7 months, respectively (p=0.719). Among the 84 EGFR-negative patients with disease progression, 8 patients were subsequently treated with immune checkpoint inhibitors, and only 3 of the 8 patients died at the last follow-up (**Figure 3**).

## Discussion

We found that the EGFR mutation-positive rate of Chinese female lung SCC patients was 29.9% from our data. For these female SCC patients with EGFR mutation, they could receive PFS and OS benefits from EGFR-TKI therapy compared with chemotherapy (PFS: 8.0 vs. 3.2 months, p=0.024; OS: 24.9 vs 13.9 months, p=0.020). However, the OS of EGFR mutation-positive patients was similar to that of EGFR negative patients (22.8 vs. 18.6 months, p=0.377).

From January 2013 to December 2018, a total of 4223 patients were diagnosed as advanced lung SCC in our hospital, of which 6.8% (288) were female, which was similar to the results of other studies (9.8%-27.0%) (30–32). It is well known that the incidence of SCC was closely related to smoking, but the smoking rate of Chinese women is relatively low, which was only 10.4% (16/154) from our observation (33–37). There might be some other reasons for the incidence of SCC in Chinese women. According to 2015 data from the Chinese Centers for Disease Control and Prevention, about 71.6% of non-smoking Chinese women have a long history of secondhand smoke exposure at home, at work or in a public environment (38, 39). On the other hand, stir frying, frying and deep frying are often used in Chinese cooking, which require high-temperature hot oil and also produce some chemical compounds (40, 41). Besides these possible reasons, air pollution, genetic susceptibility, and estrogen level might also be related to the incidence of lung cancer in Chinese women (42–44).

EGFR mutation rate of advanced female lung SCC was 29.9% (46/154) in our data, which was similar to the results of two previous Chinese studies (33.3% and 18.1%, respectively) and was significantly higher than that of male lung SCC (4.0%, 64/1599) (10, 11). This phenomenon has been reported in both Chinese (22.2% and 5.1%) and Russian population (14.4% and 1.8%), and EGFR mutation rate of female SCC patients in Russia seems lower than that in Chinese population (14.4% vs. 18.1-33.3%) (6, 45). The possible reasons are differences in smoking rate and race.

The observed mutation rates of EGFR were 17.9% (7/39) in surgical specimens and 33.9% (39/115) in small biopsies, respectively (p=0.06). It was said that EGFR mutation in patients with SCC was caused by tumor heterogeneity and the component of mixed adenocarcinoma, especially in small biopsy specimens (46). However, many studies have confirmed that EGFR mutation really occurs in patients with SCC, whether it is from small biopsy specimens or surgical specimens (7, 11).

Notably, it is very interesting to find the relationship between the exposure history of cooking oil fume and the EGFR mutation rate of Chinese female lung SCC patients. Patients with history of cooking oil fume exposure were less likely to develop EGFR positive mutations (73.9% vs. 92.6%, p=0.002), while no significant difference was observed between patients with smoking history and secondhand smoke exposure history or not. It seems long-term exposure to cooking oil fume was an important risk factor for lung cancer in Chinese women. However, the OS between the patients with the history of cooking oil fume exposure and patients without the history was similar (21.6 vs. 19.0 months, p=0.593).

The ORR and DCR of the first and second generation TKI for EGFR mutation positive non-squamous non-small cell lung cancer (NSq-NSCLC) were about 56.0%-71.2% and 90.0%-91.7%, respectively (23, 47). We found that the ORR and DCR of female patients with lung SCC treated with TKIs were 44.7% and 81.6%, respectively, which were lower than those of NSq-NSCLC, but seemed to be higher than that of patients with SCC (most of them were male patients; ORR:25.0%-31.8%, DCR: 47.3%-81.8%) (7, 9, 11, 13, 24). This suggests that although with EGFR mutation, the effect of SCC patients on TKI is not as good as that of adenocarcinoma patients, but the effect of female patients with SCC is better than that of male patients.

EGFR-positive female SCC patients achieved survival benefits from TKI treatment (PFS: 8.0 vs. 3.2 months, p=0.024; OS: 24.9 vs. 13.9 months, p=0.020). But the OS of all the EGFR-positive patients was no longer than that of EGFR-negative patients (22.8 vs. 18.6 months, p=0.377). This might be related to the small sample size of EGFR positive female lung SCC patients, the higher incidence of brain metastasis in patients with EGFR positive mutation group (21.7% and 2.8%, p < 0.001) and the use of immunotherapy of some patients as second line treatment in the EGFR-negative group, which may prolong their OS (48–51). In addition, for EGFR-positive patients, there was no significant difference between PFS (6.7 vs. 8.0 months, p=0.715) and OS (p=0.201) in first-line and second-line TKI treatment.

In NSCLC, the incidence of brain metastasis in SCC was lower than that in NSq-NSCLC (6% vs. 26.3%) (52, 53). The brain metastasis rate observed in our study in all female SCC was 8.4%. It was worth noting that the rate of brain metastasis in EGFR positive patients was much higher than that in EGFR negative patients (21.7% vs. 2.8%, p < 0.001), which was similar to the previous results of NSq-NSCLC patients (54). For EGFR mutation-positive female SCC patients, the median OS of patients with brain metastasis was significantly shorter than patients without brain metastasis (13.9 vs. 24.9 months, p=0.006). Of the 10 patients with brain metastasis and EGFR mutation positive, 8 received the first- or second-generation EGFR-TKIs (4 gefitinib, 2 icotinib, 1 erlotinib and 1 afatinib), but only 3 received third-generation TKI (osimertinib) beyond disease progression and only 2 received brain radiotherapy. This might because the number of patients with brain metastases was too small and the patients included in this study were all before 2019, the third generation of TKI was not widely used due to the high cost in China.

Because our study started early, only 25.8% (8/31) patients received re-biopsy beyond EGFR-TKI resistance, 2 cases were found the acquired T790M mutation (2/8, 25.0%), 1 case was MET amplification (1/8, 12.5%), and others without useful explicit second mutations. According to the literature, there is no report on the incidence of T790M secondary mutation after TKI in patients with SCC, but it seems to be lower than that in patients with adenocarcinoma (55). Of course, we need a larger sample size to understand this situation.

The positive rates of EGFR mutation tested by NGS and ARMS were similar (30.0% vs. 29.9%, p=0.99). NGS technology is an important detection method widely used in the precise treatment of lung cancer. It can detect multiple genes with high-throughput, and can help to find the coexisting mutations, and even subdivide the gene mutation population and help to clarify the drug resistance mechanism (56, 57). However, all the patients enrolled in our study were before 2019, when the application of NGS in China was just beginning. Therefore, it is a pity that the proportion of patients with NGS detection in this study was not high. In the article just published by our team, we analyzed the results of all female SCC patients who underwent NGS in our hospital from 2018 to 2019 and found that female patients had a high frequency of currently known or potentially actionable oncogenic alterations could benefit from targeted therapies. By comparing the NGS results of female non-smoking SCC patients and male SCC patients in TCGA database, we found that there may be significant differences in the compositions of genomic alterations of lung SQCC between female non-smokers and male smokers (27).

It must be mentioned that there were some limitations in our study. Although this is by far the largest case number study of EGFR mutations in Chinese female lung SCC, it’s still a single-center retrospective study. Due to the problem of drug accessibility, many mutation positive patients did not undergo re-biopsy to understand the mechanism of drug resistance, and lost the opportunity of three generations of TKI, unable to provide us with more in-depth information. Therefore, a prospective large-scale trial is expected to further understand the EGFR mutation rate and response to TKIs in advanced Chinese female lung SCC.

## Conclusion

In general, we have reported a large case number of Chinese female patients with lung SCC and their EGFR mutation rate and responses to TKIs. The EGFR mutation rate was 29.9% for Chinese female lung SCC patients. Patients with positive EGFR mutation could get better PFS and OS by TKI therapy, but compared with EGFR negative mutation patients, they obtained similar OS. Overall, this study has improved our understanding of female SCC and laid the foundation for us to further understand this rare population.

## Data Availability Statement

The original contributions presented in the study are included in the article/**Supplementary Material**. Further inquiries can be directed to the corresponding author.

## Ethics Statement

The studies involving human participants were reviewed and approved by The Ethics Committee of Shanghai Chest Hospital. The patients/participants provided their written informed consent to participate in this study.

## Author Contributions

Conception and design of the study: TC. Acquisition of clinical data: QC, HQ, JQ, and YL. Analysis and interpretation of the data: QC, YaZ, BH, JL, HF, and YiZ. Manuscript drafting and revision: QC and TC. All authors contributed to the article and approved the submitted version.

## Funding 

This work was supported by the Western Medicine Guide Project of Shanghai Committee of Science and Technology (Grant No.18411968500).

## Conflict of Interest

The authors declare that the research was conducted in the absence of any commercial or financial relationships that could be construed as a potential conflict of interest.

## References

[1] Rebecca LS Kimberly DM Ahmedin J. Cancer statistics, 2020. CA Cancer J Clin (2020) 70(1):7–30. 10.3322/caac.21590 31912902

[2] Chen WQ Zheng RS Peter D Zhang SW Zeng HM Freddie B. Cancer statistics in China, 2015. CA Cancer J Clin (2016) 66(2):115–32. 10.3322/caac.21338 26808342

[3] Freddie B Jacques F Isabelle S Rebecca LS Lindsey AT Ahmedin J. Global cancer statistics 2018: GLOBOCAN estimates of incidence and mortality worldwide for 36 cancers in 185 countries. CA Cancer J Clin (2018) 68(6):394–424. 10.3322/caac.21492 30207593

[4] Sarah W Camille M Michel PC Peake MD Butler J Young N. Lung cancer survival and stage at diagnosis in Australia, Canada, Denmark, Norway, Sweden and the UK: a population-based study, 2004-2007. Thorax (2013) 68(6):551–64. 10.1136/thoraxjnl-2012-202297 23399908

[5] Rafael R Teresa M Cristina Q Porta R Cardenal F Camps C. Screening for epidermal growth factor receptor mutations in lung cancer. N Engl J Med (2009) 361(10):958–67. 10.1056/NEJMoa0904554 19692684

[6] Evgeny NI Irina AD Marat GG Filipenko ML Kekeyeva TV Moliaka YK. Distribution of EGFR Mutations in 10,607 Russian Patients with Lung Cancer. Mol Diagn Ther (2016) 20(4):401–6. 10.1007/s40291-016-0213-4 27259329

[7] Fang W Zhang J Liang W Huang Y Yan Y Wu X. Efficacy of epidermal growth factor receptor-tyrosine kinase inhibitors for Chinese patients with squamous cell carcinoma of lung harboring EGFR mutation. J Thorac Dis (2013) 5(5):585–92. 10.3978/j.issn.2072-1439.2013.09.15 PMC3815724 24255770

[8] Fiala O Pesek M Finek J Benesova L Bortlicek Z Minarik M. Gene mutations in squamous cell NSCLC: insignificance of EGFR, KRAS and PIK3CA mutations in prediction of EGFR-TKI treatment efficacy. Anticancer Res (2013) 33(4):1705–11. 0250-7005/2013$2.00+.40 23564819

[9] Hata A Katakami N Yoshioka H Kunimasa K Fujita S Kaji R. How sensitive are epidermal growth factor receptor-tyrosine kinase inhibitors for squamous cell carcinoma of the lung harboring EGFR gene-sensitive mutations? J Thorac Oncol (2013) 8(1):89–95. 10.1097/JTO.0b013e31827690b5 23242440

[10] Zhang H Yang X Qin N Li X Yang H Nong J. Detection and Analysis of EGFR and KRAS Mutations in the Patients with Lung Squamous Cell Carcinomas. Chin J Lung Cancer (2015) 18(10):621–5. 10.3779/j.issn.1009-3419.2015.10.04 PMC6000093 26483334

[11] Zhou S Wang H Jiang W Yu Q. EGFR Clinicopathological Characteristics And EGFR-TKIs Efficacies In Lung Squamous Cell Carcinoma Patients Harboring An Sensitizing Mutation. Onco Targets Ther (2019) 12:8863–71. 10.2147/ott.s225760 PMC6826177 31802898

[12] Sun Y Yin X Wen MM Zhang J Wang XJ Xia JH. EGFR mutations subset in Chinese lung squamous cell carcinoma patients. Mol Med Rep (2018) 17(6):7575–84. 10.3892/mmr.2018.8859 PMC5983943 29620244

[13] Amit J Saurabh Z Vanita N Patil VM Chougule A Kumar R. EGFR mutation in squamous cell carcinoma of the lung: does it carry the same connotation as in adenocarcinomas? Onco Targets Ther (2017) 10:1859–63. 10.2147/ott.s125397 PMC5378442 28405166

[14] Yuri T Yoko M Ryutaro F Sayaka O Kazuhiro U. The clinical features of squamous cell lung carcinoma with sensitive EGFR mutations. Int J Clin Oncol (2018) 23(3):452–7. 10.1007/s10147-017-1233-8 29446042

[15] Inoue A Kobayashi K Maemondo M Sugawara S Oizumi S Isobe H. Updated overall survival results from a randomized phase III trial comparing gefitinib with carboplatin-paclitaxel for chemo-naive non-small cell lung cancer with sensitive EGFR gene mutations (NEJ002). Ann Oncol (2013) 24(1):54–9. 10.1093/annonc/mds214 22967997

[16] Mitsudomi T Morita S Yatabe Y Negoro S Okamoto I Tsurutani J. Gefitinib versus cisplatin plus docetaxel in patients with non-small-cell lung cancer harbouring mutations of the epidermal growth factor receptor (WJTOG3405): an open label, randomised phase 3 trial. Lancet Oncol (2010) 11(2):121–8. 10.1016/s1470-2045(09)70364-x 20022809

[17] Fukuoka M Wu YL Thongprasert S Sunpaweravong P Leong SS Sriuranpong V. Biomarker analyses and final overall survival results from a phase III, randomized, open-label, first-line study of gefitinib versus carboplatin/paclitaxel in clinically selected patients with advanced non-small-cell lung cancer in Asia (IPASS). J Clin Oncol (2011) 29(21):2866–74. 10.1200/JCO.2010.33.4235 21670455

[18] Han JY Park K Kim SW Lee DH Kim HY Kim HT. First-SIGNAL: first-line single-agent iressa versus gemcitabine and cisplatin trial in never-smokers with adenocarcinoma of the lung. J Clin Oncol (2012) 30(10):1122–8. 10.1200/JCO.2011.36.8456 22370314

[19] Rafael R Enric C Radj G Vergnenegre A Massuti B Felip E. Erlotinib versus standard chemotherapy as first-line treatment for European patients with advanced EGFR mutation-positive non-small-cell lung cancer (EURTAC): a multicentre, open-label, randomised phase 3 trial. Lancet Oncol (2012) 13(3):239–46. 10.1016/s1470-2045(11)70393-x 22285168

[20] Shi YK Wang L Han BH Li W Yu P Liu YP. First-line icotinib versus cisplatin/pemetrexed plus pemetrexed maintenance therapy for patients with advanced EGFR mutation-positive lung adenocarcinoma (CONVINCE): a phase 3, open-label, randomized study. Ann Oncol (2017) 28(10):2443–50. 10.1093/annonc/mdx359 28945850

[21] Wu YL Zhou CC Liam CK Wu G Liu X Zhong Z. First-line erlotinib versus gemcitabine/cisplatin in patients with advanced EGFR mutation-positive non-small-cell lung cancer: analyses from the phase III, randomized, open-label, ENSURE study. Ann Oncol (2015) 26(9):1883–9. 10.1093/annonc/mdv270 26105600

[22] Zhou CC Wu YL Chen GY Feng J Liu XQ Wang C. Erlotinib versus chemotherapy as first-line treatment for patients with advanced EGFR mutation-positive non-small-cell lung cancer (OPTIMAL, CTONG-0802): a multicentre, open-label, randomised, phase 3 study. Lancet Oncol (2011) 12(8):735–42. 10.1016/s1470-2045(11)70184-x 21783417

[23] Sequist LV Yang CH Yamamoto N O’Byrne K Hirsh V Mok T. Phase III study of afatinib or cisplatin plus pemetrexed in patients with metastatic lung adenocarcinoma with EGFR mutations. J Clin Oncol (2013) 31(27):3327–34. 10.1200/jco.2012.44.2806 23816960

[24] Xu JL Chu TQ Jin B Dong X Lou Y Zhang XY. Epidermal Growth Factor Receptor Tyrosine Kinase Inhibitors in Advanced Squamous Cell Lung Cancer. Clin Lung Cancer (2016) 17(4):309–14. 10.1016/j.cllc.2015.11.009 26725853

[25] Goldstraw P Chansky K Crowley J Rami-Porta R Asamura H Eberhardt WE. The IASLC Lung Cancer Staging Project: Proposals for Revision of the TNM Stage Groupings in the Forthcoming (Eighth) Edition of the TNM Classification for Lung Cancer. J Thorac Oncol (2016) 11(1):39–51. 10.1016/j.jtho.2015.09.009 26762738

[26] Yang W Qian F Teng J Wang H Manegold C Pilz LR. Community-based lung cancer screening with low-dose CT in China: Results of the baseline screening. Lung Cancer (2018) 117:20–6. 10.1016/j.lungcan.2018.01.003 29496251

[27] Zhao YM Dong Y Zhao RY Zhang B Wang SY Zhang LL. Expression Profiling of Driver Genes in Female Never-smokers With Non-adenocarcinoma Non-small-cell Lung Cancer in China. Clin Lung Cancer (2020) 215(5):e355–62. 10.1016/j.cllc.2020.02.005 32139332

[28] Zhang J Xiang C Han YC Teng HH Li XJ Shao JC. Differential diagnosis of pulmonary enteric adenocarcinoma and metastatic colorectal carcinoma with the assistance of next-generation sequencing and immunohistochemistry. J Cancer Res Clin Oncol (2019) 145(1):269–79. 10.1007/s00432-018-2788-0 PMC11810349 30415301

[29] Eisenhauer EA Therasse P Bogaerts J Schwartz LH Sargent D Ford R. New response evaluation criteria in solid tumours: revised RECIST guideline (version 1.1). Eur J Cancer (2009) 45(2):228–47. 10.1016/j.ejca.2008.10.026 19097774

[30] Sakurai H Asamura H Goya T Eguchi K Nakanishi Y Sawabata N. Survival differences by gender for resected non-small cell lung cancer: a retrospective analysis of 12,509 cases in a Japanese Lung Cancer Registry study. J Thorac Oncol (2010) 5(10):1594–601. 10.1097/JTO.0b013e3181f1923b 20736855

[31] Wang BY Huang JY Chen HC Lin CH Lin SH Hung WH. The comparison between adenocarcinoma and squamous cell carcinoma in lung cancer patients. J Cancer Res Clin Oncol (2020) 146(1):43–52. 10.1007/s00432-019-03079-8 31705294 PMC11804334

[32] Hendriks LE Derks JL Postmus PE Damhuis RA Houben RM Troost EG. Single organ metastatic disease and local disease status, prognostic factors for overall survival in stage IV non-small cell lung cancer: Results from a population-based study. Eur J Cancer (Oxford Engl 1990) (2015) 51(17):2534–44. 10.1016/j.ejca.2015.08.008 26323530

[33] Auerbach O Stout AP Hammond EC Garfinkel L. Changes in bronchial epithelium in relation to cigarette smoking and in relation to lung cancer. N Engl J Med (1961) 265:253–67. 10.1056/nejm196108102650601 13685078

[34] Kim HR Kim DJ Kang DR Lee JG Lim SM Lee CY. Fibroblast growth factor receptor 1 gene amplification is associated with poor survival and cigarette smoking dosage in patients with resected squamous cell lung cancer. J Clin Oncol (2013) 31(6):731–7. 10.1200/jco.2012.43.8622 23182986

[35] Freedman ND Leitzmann MF Hollenbeck AR Schatzkin A Abnet CC. Cigarette smoking and subsequent risk of lung cancer in men and women: analysis of a prospective cohort study. Lancet Oncol (2008) 9(7):649–56. 10.1016/s1470-2045(08)70154-2 PMC2601691 18556244

[36] Hirayama T. Passive smoking and lung cancer: consistency of association. Lancet (1983) 2(8364):1425–6. 10.1016/s0140-6736(83)90960-1 6140528

[37] Zeng Q Vogtmann E Jia MM Parascandola M Li JB Wu YL. Tobacco smoking and trends in histological subtypes of female lung cancer at the Cancer Hospital of the Chinese Academy of Medical Sciences over 13 years. Thorac Cancer (2019) 10(8):1717–24. 10.1111/1759-7714.13141 PMC6669800 31293059

[38] Nan Y Wang LL Chen XY Feng GZ Yang Y. Analysis of Chinese women’s awareness of the hazards of smoking and secondhand smoke and their exposure to secondhand smoke. Chin J Prev Control Chronic Dis (2015) 23(6):443–5. 10.16386/j.cjpccd.issn.1004-6194.2015.06.011

[39] Bach PB. Smoking as a factor in causing lung cancer. JAMA (2009) 301(5):539–41. 10.1001/jama.2009.57 19190320

[40] Chen TY Fang YH Chen HL Chang CH Huang H Chen YS. Impact of cooking oil fume exposure and fume extractor use on lung cancer risk in non-smoking Han Chinese women. Sci Rep (2020) 10(1):6774. 10.1038/s41598-020-63656-7 32317677 PMC7174336

[41] Lee T Gany F. Cooking oil fumes and lung cancer: a review of the literature in the context of the U.S. population. J Immigr Minor Health (2013) 15(3):646–52. 10.1007/s10903-012-9651-1 22678304

[42] Hamra GB Guha N Cohen A Laden F Ole RN Samet JM. Outdoor Particulate Matter Exposure and Lung Cancer: A Systematic Review and Meta-Analysis. Environ Health Perspect (2014) 122(9):906–11. 10.1016/j.envpol.2020.115328 PMC4154221 24911630

[43] Lan Q Hsiung CA Matsuo K Hong YC Seow A Wang Z. Genome-wide association analysis identifies new lung cancer susceptibility loci in never-smoking women in Asia. Nat Genet (2012) 44(12):1330–5. 10.1038/ng.2456 PMC4169232 23143601

[44] Pesatori AC Carugno M Consonni D Hung RJ Papadoupolos A Landi MT. Hormone use and risk for lung cancer: a pooled analysis from the International Lung Cancer Consortium (ILCCO). Br J Cancer (2013) 109(7):1954–64. 10.1038/bjc.2013.506 PMC3790162 24002594

[45] Yu QT Qi LF Zeng AP Song XQ Zhou SZ. Status of EGFR Gene Mutation in Lung Squamous Cell Carcinoma and Efficacy of EGFR-TKIs. J Chin Oncol (2019) 25(5):405–8. 10.11735/j.issn.1671-170X.2019.05.B005

[46] Rekhtman N Paik PK Arcila ME Tafe LJ Oxnard GR Moreira AL. Clarifying the spectrum of driver oncogene mutations in biomarker-verified squamous carcinoma of lung: lack of EGFR/KRAS and presence of PIK3CA/AKT1 mutations. Clin Cancer Res (2012) 18(4):1167–76. 10.1158/1078-0432.ccr-11-2109 PMC3487403 22228640

[47] Mok TS Wu YL Thongprasert S Yang CH Chu DT Saijo N. Gefitinib or carboplatin-paclitaxel in pulmonary adenocarcinoma. N Engl J Med (2009) 361(10):947–57. 10.1056/NEJMoa0810699 19692680

[48] Brahmer J Reckamp KL Baas P Crinò L Eberhardt WE Poddubskaya E. Nivolumab versus Docetaxel in Advanced Squamous-Cell Non-Small-Cell Lung Cancer. N Engl J Med (2015) 373(2):123–35. 10.1056/NEJMoa1504627 PMC4681400 26028407

[49] Paz-Ares L Luft A Vicente D Tafreshi A Gümüş M Mazières J. Pembrolizumab plus Chemotherapy for Squamous Non-Small-Cell Lung Cancer. N Engl J Med (2018) 379(21):2040–51. 10.1056/NEJMoa1810865 30280635

[50] Reck M Rodríguez-Abreu D Robinson AG Hui R Csőszi T Fülöp A. Pembrolizumab versus Chemotherapy for PD-L1-Positive Non-Small-Cell Lung Cancer. N Engl J Med (2016) 375(19):1823–33. 10.1056/NEJMoa1606774 27718847

[51] Mok TS Wu YL Kudaba I Kowalski DM Cho BC Turna HZ. Pembrolizumab versus chemotherapy for previously untreated, PD-L1-expressing, locally advanced or metastatic non-small-cell lung cancer (KEYNOTE-042): a randomised, open-label, controlled, phase 3 trial. Lancet (2019) 393(10183):1819–30. 10.1016/s0140-6736(18)32409-7 30955977

[52] Goncalves PH Peterson SL Vigneau FD Shore RD Quarshie WO Islam K. Risk of brain metastases in patients with nonmetastatic lung cancer: Analysis of the Metropolitan Detroit Surveillance, Epidemiology, and End Results (SEER) data. Cancer (2016) 122(12):1921–7. 10.1002/cncr.30000 PMC4892979 27062154

[53] Hsu F De Caluwe A Anderson D Nichol A Toriumi T Ho C. EGFR mutation status on brain metastases from non-small cell lung cancer. Lung Cancer (2016) 96:101–7. 10.1016/j.lungcan.2016.04.004 27133758

[54] Li L Luo SM Lin H Yang HT Chen HJ Liao ZY. Correlation between EGFR mutation status and the incidence of brain metastases in patients with non-small cell lung cancer. J Thorac Dis (2017) 9(8):2510–20. 10.21037/jtd.2017.07.57 PMC5594201 28932557

[55] Chee-Seng T David G Simon P. Treatment approaches for EGFR-inhibitor-resistant patients with non-small-cell lung cancer. Lancet Oncol (2015) 16(9):e447–e59. 10.1016/s1470-2045(15)00246-6 26370354

[56] Goodwin S McPherson JD McCombie WR. Coming of age: ten years of next-generation sequencing technologies. Nat Rev Genet (2016) 17(6):333–51. 10.1038/nrg.2016.49 PMC10373632 27184599

[57] Cardarella S Ortiz TM Joshi VA Butaney M Jackman DM Kwiatkowski DJ. The introduction of systematic genomic testing for patients with non-small-cell lung cancer. J Thorac Oncol (2012) 7(12):1767–74. 10.1097/JTO.0b013e3182745bcb PMC3500523 23154547

