# Supplementary material for: Epidermal Growth Factor Receptor Mutation Status and Response to Tyrosine Kinase Inhibitors in Advanced Chinese Female Lung Squamous Cell Carcinoma: A Retrospective Study
Source: Front Oncol. 2021 Apr 2;11:652560. doi: 10.3389/fonc.2021.652560 (PMC8050333; doi:10.3389/fonc.2021.652560)
Supplement: Supplementary file 1 [file Table_1.docx]

Supplementary table 1. The clinical characteristics of patients with treated

| **Characteristics** | **All patients**  (n=150) | | | **p-value** | |
| --- | --- | --- | --- | --- | --- |
|  | **Mutation-positive** (n=45) | **Mutation-negative** (n=105) | |  |  |
| Median age, years | 59.9 | | 59.2 | | 0.967 |
| Smoking history |  | |  | | 0.555 |
| Former smoker | 3(6.7%) | | 11(10.5%) | |  |
| Never smoker | 42(93.3%) | | 94(89.5%) | |  |
| Long history of exposure to secondhand smoke |  | |  | | 0.590 |
| Yes | 27(60.0%) | | 58(55.2%) | |  |
| No | 18(40.0%) | | 47(44.8%) | |  |
| Long history of exposure to cooking oil fume |  | |  | | **<0.001** |
| Yes | 33(73.3%) | | 99(94.3%) | |  |
| No | 12(26.7%) | | 6(5.7%) | |  |
| Mutation status |  | |  | | - |
| Exon 19 deletion | 23(51.1%) | | - | |  |
| L858R mutation | 20(44.4%) | | - | |  |
| Others | 2(4.4%) | | - | |  |
| Tumor location |  | |  | | 0.299 |
| Central type | 23(51.1%) | | 61(58.1%) | |  |
| Peripheral type | 22(48.9%) | | 44(41.9%) | |  |
| Tumor stage |  | |  | | 0.625 |
| ⅢB | 14(31.1%) | | 37(35.2%) | |  |
| Ⅳ | 31(68.9%) | | 68(64.8%) | |  |
| Brain metastasis |  | |  | | **<0.001** |
| Yes | 10(22.2%) | | 3(2.9%) | |  |
| No | 35(77.8%) | | 102(97.1%) | |  |
| Specimen |  | |  | | 0.056 |
| Operation | 7(15.6%) | | 32(30.5%) | |  |
| Small biopsy | 38(84.4%) | | 73(69.5%) | |  |

Abbreviations: EGFR, epidermal growth factor receptor; TKI, tyrosine-kinase inhibitor.

Two-sided P value was derived from Wilcoxon rank sum test for Continuous variables and from Chi-square test or Fisher’s exact test for Categorical variables.
